# Temporal orienting in Parkinson’s disease

**DOI:** 10.1111/ejn.15114

**Published:** 2021-02-02

**Authors:** Nahid Zokaei, Celine R. Gillebert, Joshua J. Chauvin, Daniela Gresch, Alexander G. Board, Michal Rolinski, Michele T. Hu, Anna Christina Nobre

**Affiliations:** ^1^ Oxford Centre for Human Brain Activity Wellcome Centre for Integrative Neuroimaging Department of Psychiatry University of Oxford Oxford UK; ^2^ Department of Experimental Psychology University of Oxford Oxford UK; ^3^ Department of Brain and Cognition KU Leuven Leuven Belgium; ^4^ Translational Health Sciences University of Bristol Bristol UK; ^5^ Department of Neurology Nuffield Department of Clinical Neurosciences University of Oxford Oxford UK

**Keywords:** attention, expectation, Parkinson's disease, RSVP, temporal orienting

## Abstract

Temporal orienting of attention can affect multiple stages of processing to guide adaptive behaviour. We tested whether temporal expectation in different task contexts is compromised in individuals with Parkinson's disease (PD). In Experiment 1 two temporal‐orienting tasks were used: a speeded task emphasizing motor preparation and a non‐speeded task emphasizing perceptual discrimination using rapid serial visual presentation. In both tasks, auditory cues indicated the likelihood of a target appearing after a short or long interval. In the speeded‐response task, participants used the cues to anticipate an easily detectable target stimulus. In the non‐speeded perceptual‐discrimination task, participants used the cues to help discriminate a target letter embedded in a stream of letters. Relative to healthy participants, participants with PD did not show altered temporal orienting effects in the speeded‐response task. However, they were impaired in using temporal cues to improve perceptual discrimination. In Experiment 2, we tested whether the temporal‐orienting deficits in the perceptual‐discrimination task depended on the requirement to ignore temporally distracting stimuli. We replicated the impaired temporal orienting for perceptual discrimination in an independent group of individuals with PD, and showed the impairment was abolished when individuals were on their dopaminergic medication. In a task without any distracting letters, however, patients off or on medication benefited normally from temporal orienting cues. Our findings suggest that deficits in temporal orienting in individuals with PD interact with specific task demands, such as the requirement to select target from temporally competing distractors.

## Graphical abstract

Temporal orienting of attention can affect multiple stages of processing to guide adaptive behaviour. We tested whether temporal expectation in different task contexts is compromised in individuals with Parkinson’s disease (PD). In Experiment 1 two temporal‐orienting tasks were used: a speeded task emphasizing motor preparation and a non‐speeded task emphasizing perceptual discrimination using rapid serial visual presentation (RSVP). In both tasks, auditory cues indicated the likelihood of a target appearing after a short or long interval. In the speeded‐response task, participants used the cues to anticipate an easily detectable target stimulus. In the non‐speeded perceptual‐discrimination task, participants used the cues to help discriminate a target letter embedded in a stream of letters. Relative to healthy participants, participants with PD did not show altered temporal orienting effects in the speeded‐response task. However, they were impaired in using temporal cues to improve perceptual discrimination. In Experiment 2, we tested whether the temporal‐orienting deficits in the perceptual‐discrimination task depended on the requirement to ignore temporally distracting stimuli. We replicated the impaired temporal orienting for perceptual discrimination in an independent group of individuals with PD, and showed the impairment was abolished when individuals were on their dopaminergic medication. In a task without any distracting letters, however, patients off or on medication benefited normally from temporal orienting cues. Our findings suggest that deficits in temporal orienting in individuals with PD interact with specific task demands, such as the requirement to select target from temporally competing distractors.

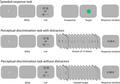

## 1 INTRODUCTION

The utilization of temporal structure in incoming stimulation to guide adaptive behaviour is a pervasive brain function (Nobre & van Ede, 2018). A variety of temporal predictions have been shown to benefit behaviour, such as specific temporal associations between successive stimuli and temporal rhythms. Temporal expectations can affect multiple stages of processing, from early perceptual analysis of stimuli to motor preparation and execution (see Nobre & van Ede, 2018; Nobre & Rohenkohl, 2014).

The brain sources of temporal expectations, however, are not yet well‐understood. Neuroimaging studies have revealed networks of brain regions whose activity varies with temporal orienting of attention (Coull & Nobre, 1998, 2008; Nobre & Rohenkohl, 2014). In particular, activity in the left inferior posterior parietal cortex and associated frontal regions has been repeatedly noted (Coull et al., 2016; Coull & Nobre, 2008; Nobre & Rohenkohl, 2014). Neuroimaging, however, has limitations in defining the functional specialization of brain areas (see Nobre & van Ede, 2020). For example, it can be difficult to separate brain areas involved in generating temporal expectations from those that are modulated as a consequence. Other contributing areas may be missed altogether if their patterns, but not levels, of activation change within tasks (Nobre & O’Reilly, 2004).

Studies testing for causal involvement of brain networks or brain areas in temporal expectation are still few in number and do not yet provide a clear consensus. One emerging insight is that behavioural benefits from different types of predictable temporal structures may rely on dissociable brain systems and mechanisms. For example, right frontal lesions disrupt the benefits of temporal orienting brought about by informative temporal cues (Triviño et al., 2010), but not by a rhythmic context (Triviño et al., 2011). Benefits from temporal cues versus rhythms have also been found to be differentially susceptible to cerebellar versus basal‐ganglia damage. Individuals with cerebellar neurodegeneration showed no benefit from temporal cues but benefitted normally from temporal rhythms; whereas individuals with Parkinson's disorder showed the complementary pattern (Breska & Ivry, 2018). In addition, dissociations have also been noted between the ability to utilize temporal structure to benefit behavioural performance implicitly versus the ability to perceive or reproduce temporal intervals explicitly (Coull & Nobre, 2008; Mioni et al., 2018; Triviño et al., 2010).

In understanding the neural systems required to support temporal orienting of attention, it may be important to consider not only the nature of the temporal prediction guiding information processing (e.g., cues or rhythms), but also the purpose of the task (Shalev et al., 2019). Sources of temporal expectation may, in principle, develop or express themselves locally within task‐specific networks that are shaped by the perceptual, cognitive and motor demands.

In the current study, we conducted two experiments to test the ability of individuals with Parkinson's disease (PD) to benefit from temporal cues under different conditions of task demands, which emphasized either speeded action or perceptual sensitivity. The nigrostriatal system is the primary target in PD, thus compromising activity in the basal ganglia and associated frontal areas (Braak et al., 2003; Koller, 1992). In addition, as the disease progresses, other major brain networks become affected and lead to variable deficits in cognition, mood, impulse control, psychosis and sleep (e.g., Gratwicke et al., 2015; Jahanshahi et al., 2015; Weintraub & Mamikonyan, 2019). The central role played by the basal ganglia and their cortical connections in regulating cognition (Alexander et al., 1986; Middleton & Strick, 2002; Stephenson‐Jones et al., 2012) place them in a privileged position to learn temporal structures and utilize them to influence perceptual and motor processing. Relevant to a putative role in participating in temporal expectation, they are involved not only in motor control (Alexander et al., 1986), but also in incremental probabilistic learning (Packard & Knowlton, 2002; Yin & Knowlton, 2006), skill learning (Charlesworth et al., 2012; Foerde & Shohamy, 2011), inhibition (McNab & Klingberg, 2008), motivation (Braunlich & Seger, 2013; Middleton & Strick, 2002), visuospatial attention (Karnath et al., 2002; Mesulam, 1990) and in timing mechanisms (Allman & Meck, 2012; Allman et al., 2014; Merchant et al., 2013; Teki et al., 2011). Yet, surprisingly, deficits in temporal cueing have not been observed in individuals with PD (Breska & Ivry, 2018; Mioni et al., 2018) or basal‐ganglia lesions (Triviño et al., 2010) thus far, despite clear deficits in explicit timing tasks (see Jones & Jahanshahi, 2014; Merchant et al., 2008, 2013; Mioni et al., 2018).

We revisited the question of whether PD is associated with deficits in cue‐driven temporal expectations. Our study differs from previous investigations in testing individuals off their medication, as well as by manipulating the specific demands imposed by the task. We compared performance of two separate groups of individuals with PD relative to well‐matched control groups across two experiments, each containing two tasks. In the first experiment, we tested for benefits of temporal orienting in a speeded‐response task and in a non‐speeded perceptual‐discrimination task (Chauvin et al., 2016). Here, we sought to examine whether temporal orienting of attention will be differentially influenced when benefiting an action versus perception in the context of PD, which has predominantly motor deficits at its core. In the second experiment, we followed up the impairment in temporal orienting of attention in the non‐speeded perceptual discrimination task in patients with PD by comparing the effects of temporal orienting in tasks with and without distractors. This was done to identify whether lack of temporal orienting is dependent upon the requirement to suppress distractors in PD, considering that patients with PD, in general, have deficits in distractibility. Therefore, finding dissociable patterns, depending on task demands and context, would refine the evidence regarding impairments of temporal orienting in PD patients as well as that regarding the principles governing specialized networks supporting temporal expectation.

## 2 EXPERIMENT 1: IMPAIRED TEMPORAL ORIENTING IN A PERCEPTUAL‐DISCRIMINATION TASK IN PARKINSON'S DISEASE

### 2.1 Materials and Methods

#### 2.1.1 Participants

The study was approved by the Oxfordshire Research Ethics Committee as part of the National Research Ethics Service, and participants gave written informed consent in accordance to the Declaration of Helsinki. All participants reported normal or corrected‐to‐normal vision.

Eighteen individuals with idiopathic PD and 18 age‐ and education‐matched healthy controls participated in Experiment 1 (Table 1). Participants with PD were recruited with the help of the Dementias and Neurodegeneration Specialty (DeNDRoN) and fulfilled the UK PD Brain‐Bank criteria for probable PD (Hughes et al., 1992). Inclusion criteria were: having a diagnosis of PD within 5 years of the participation date and being a native English speaker. Exclusion criteria were: being an active participant in an ongoing clinical drug trial; not tolerating coming off medication; taking psychotropic, hypertensive or vasoactive medication; or having a history of neurological or psychiatric disease other than PD.

**TABLE 1:** Demographics, MoCA and UPDRS scores of PD and control participants in Experiment 1

	PD participants		Control participants		Mann–Whitney *U*‐test
	Mean (*SE*)	Range	Mean (*SE*)	Range	*p*
Age (year)	68.9 (1.6)	54–79	67.3 (1.1)	60–76	*n*.s.
Education (year)	13.8 (0.8)	10–23	15.1 (0.9)	10–20	*n*.s.
MoCA	26.2 (0.7)	18–29	28.3 (0.3)	26–30	<.05
UPDRS – III	33.2 (4.01)	11–81	1.3 (0.4)	0–4	<.001

Participants with PD were asked to withdraw from their dopaminergic medication at 7 p.m. the night before the experiments. The duration of participants being off their medication is comparable to other studies investigating the effects of PD on timing‐related experiments (e.g., O’Boyle et al., 1996). Control participants were recruited from the Oxford Dementia and Ageing Research database and were required to have a Montreal Cognitive Assessment (MoCA) score equal to or above 26 (Nasreddine et al., 2005). In addition, both PD and control participants were examined by a trained clinician using the Unified Parkinson's Disease Rating Scale (UPDRS) (Goetz et al., 2008) in order to verify and quantify deficits in the Parkinson's group. The UPDRS‐III Motor Examination was administered when participants were off medication (Table 1).

#### 2.1.2 Stimuli and task procedure

Stimulus presentation and response collection were controlled by an ASUS PC Notebook running Presentation 16.5 (Neurobehavioural systems, Albany, CA, USA). Participants sat in a dimly lit room approximately 55 cm from a 15.4‐inch monitor (resolution 1,440 × 900 pixels, refresh rate 60 Hz). The sampling rate for responses was set at 8ms.

**FIGURE 1:**
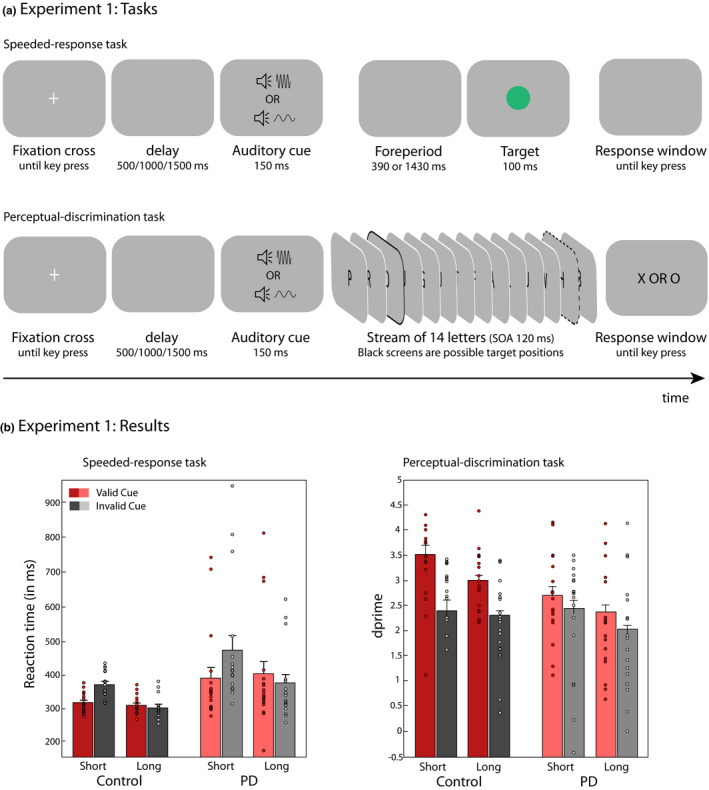
(a) Schematic illustration of the speeded‐response task and the perceptual‐discrimination task. Auditory cues predicted when target events were more likely to occur. Speeded‐response task: Targets consisted of green circular patches presented on each trial foveally 540 or 1580 ms after the cue onset (stimulus‐onset interval). Participants were instructed to respond as quickly as possible to the green patch by pressing the left arrow key on a standard keyboard with their right index finger. Perceptual‐discrimination task: Targets were either an X or an O presented foveally at 540 or 1620 ms within the visual stream. The two foreperiods are indicated by a black outline. Participants were instructed to hold off on responding until the end of the trial, and to press the left arrow key if they thought they saw an X and the right arrow key if they thought they saw an O. (b) Temporal orienting effects in participants with Parkinson’s disease (PD) and healthy control participants for targets appearing after a short and long interval, for expected (valid) and unexpected (invalid) targets; for the speeded response (left panel) and the perceptual‐discrimination (right panel) tasks. Error bars represent standard errors of the means (SEM)

The experiment consisted of a speeded‐response task and a non‐speeded perceptual‐discrimination task (based on Chauvin et al., 2016). In the speeded‐response task (Figure 1a, top panel), participants were instructed to respond as quickly as they could to a green circular patch, which appeared on each trial at the center of the screen. In the non‐speeded perceptual‐discrimination task (Figure 1a, bottom panel), participants were instructed to identify a target letter (‘X’ or ‘O’) embedded in a stream of 14 rapidly presented distractor letters and to provide a non‐speeded response at the end of the trial. In both tasks, the pitch of an auditory cue preceding the target, 1,100 or 600 Hz, indicated the likelihood of the target item occurring after a short or long temporal interval, respectively. The cue was valid in 75% of the trials. Participants were instructed to maintain central fixation and to use the predictive temporal information provided by the cue to anticipate the occurrence of the target.

Stimuli appeared superimposed against a uniform grey background (RGB values: 128, 128, 128), and a fixation point remained visible in the centre of the screen. Each trial commenced following a participant‐initiated key press. After a short delay lasting 500 ms (50% probability), 1,000 ms (25% probability) or 1,500 ms (25% probability), an audio cue was presented for 150 ms. In the speeded‐response task, participants were asked to respond as quickly as possible with their right index finger upon detecting a centrally presented green target patch, occurring after a stimulus‐onset asynchrony (SOA) of 540 ms or 1,580 ms. In the non‐speeded perceptual‐discrimination task, the audio cue was followed by a stream of 14 black letters (font OCR A Extended) presented foveally and in rapid succession (100‐ms duration and 20‐ms inter‐stimulus interval). The SOA between the audio cue and the first letter was 390 ms. Thirteen letters were distractors and one letter was a target letter. The target letter, ‘X’ or ‘O’, appeared either early (on the 3rd location, after 540 ms) or late (on the 12th location, after 1,580 ms). The distracter stimuli were randomly sampled without replacement from a set of 16 letters [A,B,E,F,G,H,I,J,L,M,P,Q,R,T,U,W]. Following the presentation of the letter stream, participants made a delayed discrimination response using their right hand using the left (for ‘X’) and right (for ‘O’) arrow keys on the keyboard (Figure 1a). Participants were under no time pressure to provide a response, and were informed that only the accuracy of the response would be taken into account. All participants used their right index finger. Target letter was an ‘X’ in half of the trials and ‘O’ in the remaining half. Moreover, there was no difference in performance between the two target letters of ‘X’ and ‘O’.

For each task, participants completed two blocks of 96 trials separated by a brief pause. Practice trials were given before each set of two blocks. The order of the tasks was counterbalanced across participants. The tasks took approximately 1 hr to complete.

#### 2.1.3 Analysis

For the speeded‐response task, the primary outcome variable was the mean reaction time (RT) on correct responses for each condition. Trials with RTs more than three standard deviations above the mean RT across all conditions and anticipatory responses, i.e. responses occurring before the onset of the target or responses with a RT less than 100 ms, were excluded from the analyses. The average number of outlier trials was low (<2%, 0–6 trials on average) and did not differ between the PD and healthy participants (*t*(34) = −0.4, *p* = .69). For the non‐speeded perceptual discrimination task, the primary outcome variable was perceptual sensitivity (*d’*). Sensitivity to stimulus was calculated according to the formula: *d’* = *z*[*PC_X_*] + z[*PC_O_*], where *PC_X_* and *PC_O_* correspond to the proportion of correct responses to the two target letters, respectively, and z corresponds to the inverse normal (z‐score) transformation (Rohenkohl et al., 2014). Trials were excluded from the analysis if the RT was more than three standard deviations above the mean RT across all conditions. For each measure, participants who scored more than three standard deviations away from the average value within each group in at least one condition were excluded from the analysis. The average number of outlier trials was low (<2%, 1–7 trials on average) and did not differ between PD and healthy participants (*t*(34) = −1.5, *p* = .14).

In order to examine how PD affected temporal orienting, a mixed‐effects analysis‐of‐variance (ANOVA) was conducted with foreperiod (short, long) and cue validity (valid, invalid) as within‐subjects factors, and participant group (PD participants, healthy participants) as a between‐subjects factor for each task. To exclude the possibility that group differences in temporal orienting effects could be attributed to reduced cognitive abilities in participants with PD, an analysis of covariance was conducted with the MoCA scores (mean‐centred across the two groups) included as a covariate.

### 2.2 Results

#### 2.2.1 Speeded‐response task

After removing the anticipatory responses, accuracy was at ceiling (<1% misses) for all conditions in both groups. Before analysing the between‐group differences, individual participants scoring more than 3 standard deviations (*SD*) above or below the average performance of all the other participants were removed from the analysis. As a result, one participant in the control group was excluded from the analysis for low performance.

A mixed‐effects ANOVA on the RT indicated main effects of group, cue validity, and foreperiod, as well as a foreperiod‐by‐validity interaction (all other *p*s > .19) (see Table S1 for summary statistics). The pattern of results did not change when including the MoCA scores as a covariate of no interest. Overall, both groups displayed the typical asymmetric cueing benefit for short versus long foreperiods. As expected, participants with PD were also significantly slower than control participants, but this main effect did not interact with the task manipulations (Figure 1b – left panel). Post‐hoc paired‐sample *t*‐tests were conducted to inform the foreperiod‐by‐validity interaction, which was significant within each group (control participants: *F*(1,16) = 17.48, *p *< .001, *η^2^* = 0.52; participants with PD: *F*(1,17) = 8.84, *p* < .001, *η^2^* = 0.34) (Table S1). Reaction times to targets appearing after a short foreperiod were significantly shorter when the preceding cue contained valid compared to invalid temporal information (*t*(34) = −5.55, *p* < .001). The effect size was very large in both groups (control participants: Cohen's *d* = 1.61; participants with PD: Cohen's *d* = 1.01). In contrast, the validity of the auditory cue did not significantly modulate RT to targets appearing after a long foreperiod (*t*(34) = 1.80, *p* = .08) (Figure 1b – left panel).

#### 2.2.2 Perceptual discrimination task

A mixed‐effects ANOVA on *d’* values revealed significant main effects of validity and foreperiod, as well as a significant interaction between validity and group (all other *p*s > .08) (Table S2). The pattern of results did not change when including the MoCA scores as a covariate of no interest.

Separate ANOVAs for PD and control participants were conducted to inform the validity‐by‐group interaction. In summary, while healthy control participants exhibited a perceptual advantage for validly cued information, this effect was not significant in participants with PD. The main effect of cue validity was significant in control participants (*F*(1,17) = 21.21, *p *< .001, *η^2^* = 0.56) (Table S2), who responded with greater perceptual sensitivity to the target when the preceding auditory cue contained valid information compared to invalid temporal information (Cohen's *d* = .96). In contrast, participants with PD did not respond with significantly greater sensitivity to validly cued targets than to invalidly cued targets (*F* (1,17) = 2.48, *p* = .13) (Figure 1b – right panel).

## 3 EXPERIMENT 2: BENEFITS OF TEMPORAL ORIENTING IN PD DEPENDS ON THE PRESENCE OF DISTRACTORS AND ON MEDICATION

### 3.1 Materials and methods

#### 3.1.1 Participants

The study was approved by the Oxfordshire Research Ethics Committee as part of the National Research Ethics Service, and participants gave written informed consent in accordance to the Declaration of Helsinki. All participants had normal or corrected‐to‐normal vision.

Fourteen individuals with idiopathic PD and 18 age‐ and education‐matched healthy controls participated in Experiment 2 (Table 2). Participants with PD were recruited from Memory Clinics in Oxfordshire. Inclusion and exclusion criteria were similar to Experiment 1, except that there was no limit on disease duration. Additionally, participants with PD were required to have an Addenbrooke's Cognitive Examination (ACE‐III) score of higher than 82 (Berankova et al., 2015) and to be capable of performing the tasks off their dopaminergic medication. Participants with PD were tested on two separate days (separated by a minimum of 2 days), on and off their medication. For off‐medication sessions, participants were asked to withdraw from their dopaminergic medication at 7 p.m. the night before the experiment. The order of sessions was counterbalanced in the PD group; seven participants performed the tasks off their medication in session one, while the remaining seven were on medication in their first session.

**TABLE 2:** Demographics, Addenbrookes Cognitive Examination‐III (ACE‐III) and UPDRS scores of PD and healthy control participants in Experiment 2

	PD participants		Control participants		Mann–Whitney *U*‐test
	Mean (*SE*)	Range	Mean (*SE*)	Range	*p*
Age (year)	65.7 (1.6)	52–74	68.1 (1.07)	54–76	n.s.
Education (year)	16.2 (1.05)	10–24	15.7 (0.6)	10–19	n.s.
ACE – III	96.6 (0.6)	88–100	97 (0.9)	90–100	n.s.
UPDRS – III	40.2 (7.23)	16–86	n/a	n/a	n/a
Daily Levodopa Equivalent dose	327.5 (141)	150–540	n/a	n/a	n/a

#### 3.1.2 Stimuli and task procedures

Stimulus presentation and response registration were controlled by MATLAB. Participants were seated in a dimly lit room approximately 55 cm from a 15.4‐inch monitor (resolution 1,440 × 900 pixels, refresh rate 60 Hz). The sampling rate for responses was set at 8ms.

**FIGURE 2:**
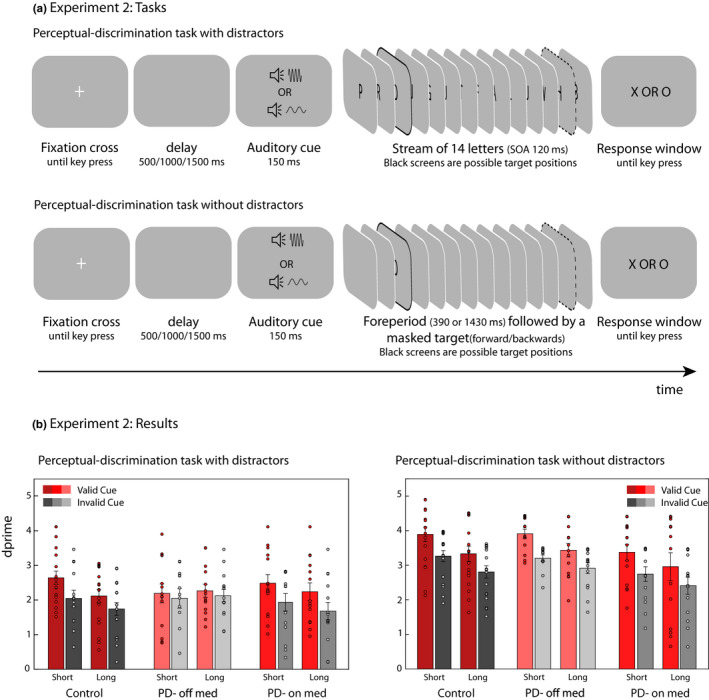
(a) Schematic illustration of the perceptual‐discrimination task with or without distractor. Auditory cues predicted when target events were more likely to occur. In the perceptual‐discrimination task targets were either an X or an O presented foveally at 540 or 1620 ms within the visual stream. The two foreperiods are indicated by a black outline. Participants were instructed to hold off on responding until the end of the trial, and to press the left arrow key if they thought they saw an X and the right arrow key if they thought they saw an O. In the task with distractors, the target items were embedded within the stream of distractor letter while the target in the perceptual task without distractors was presented masked (forward and backwards) in the absence of any distracting letter. (b) Temporal orienting effects in participants with Parkinson’s disease and healthy control participants for targets appearing after a short and long interval, for expected (valid) and unexpected (invalid) targets; for the perceptual discrimination task with (a) or without (b) distractors. Error bars represent standard errors of the means

The experiment consisted of non‐speeded perceptual‐discrimination tasks (Figure 2a), with or without distractors. The discrimination task with distractors was identical to that described in Experiment 1 except for the following changes. The letter ‘Q’ was replaced by the letter ‘Z’ as a distracter (full set: A,B,E,F,G,H,I,J,L,M,P,R,T,U,W,Z) to avoid confusion with the target letter ‘O’. Similar to Experiment 1, there was no difference in performance between the two target letters of ‘X’ and ‘O’.

In the discrimination task without distractors (Figure 2a), the auditory cue was followed by a blank delay before the presentation of the target letter, either early (after 540 ms from cue onset) or late (after 1,580 ms from cue onset). The target letter was masked, both prior and after the presentation of the target letter (inter‐stimulus interval of 20 ms), for 100 ms. The mask consisted of superimposed set of five randomly selected letters from the distractors in the discrimination task with distractors and the hash key. Crucially, participants were asked to withhold their response until the end of the trial and respond only when probed at the end of the trial, making the responding demands similar to those in the discrimination task with distractors. In both tasks, participants were under no time pressure to provide a response and were informed that only the accuracy of the response would be taken into account.

For each task, participants completed two blocks of 96 trials separated by a brief pause. Practice trials were given before each set of two blocks. The order of the tasks was counterbalanced across participants. The tasks took approximately 1 hr to complete.

The same outlier threshold as that employed in Experiment 1 was applied to Experiment 2. Trials were excluded from the analysis if the RT was more than three standard deviations above the mean RT across all conditions. The average number of outlier trials was low (<2%, 0–6 trials on average) and did not differ between PD off‐medication and healthy participants (*t*(30) = 0.27, *p* = .78) or PD participants on and off medication (*t*(13)= 0.4, *p* = .69).

### 3.2 Results

The pattern of results did not change when including the ACE scores as a covariate of no interest. For the following analyses, ACE scores were not included.

#### 3.2.1 Perceptual discrimination task with distractors

The first analysis aim was to replicate the findings from Experiment 1. A mixed‐effects ANOVA on *d′* values on PD participants off medication and healthy controls revealed a significant main effect of foreperiod and validity, as well as a significant interaction between group and validity (all other *ps* > .1) (Table S3).

Similar to Experiment 1, separate ANOVAs for off‐medication PD and control participants were conducted to inform the validity‐by‐group interaction. The main effect of validity (*F*(1,17) = 8.08, *p* = .011, *η^2^* = .312) and foreperiod (*F*(1,17) = 7.21, *p* = .016, *η^2^* = .29) was significant in control participants (Table S4). Replicating Experiment 1, healthy participants responded with higher perceptual sensitivity to validly cued targets appearing earlier in the sequence. In contrast, PD participants off‐medication showed no effect of validity, foreperiod or an interaction between the two factors (all other *ps* > .3, Figure 2b) (Table S4).

To examine the effect of dopaminergic medication on performance in this task, we next performed repeated‐measures ANOVA on *d’* values for PD participants on versus off medication. There was a significant main effect of validity and a significant interaction between validity and medication (Table S4). Follow‐up analysis revealed that, unlike when off medication, PD participants on their dopaminergic medication demonstrated a significant increase in perceptual sensitivity for validly cued targets (main effect of validity: *F*(1,13) = 10.5, *p* = .006, *η^2^* = .45).

Next, we conducted a mixed‐effects ANOVA on *d′* values on PD participants on medication and healthy controls. We observed a significant main effect of foreperiod and validity, with no effect of group or interaction between any of the factors (all other *ps* > .4) (Table S3).

Lastly, although no emphasis was placed on response times, for completeness we investigated the effect of group, medication and task conditions on response times (Tables S5 and S6). PD participants’ response times were similar to that of healthy controls and overall the PD group responded faster when off medication compared to when on medication regardless of task condition (see Tables S5 and S6). Importantly, there was a significant effect of validity on response times, highlighting that any effects on *d’* values were not just a trade‐off between response times and accuracy.

#### 3.2.2 Perceptual discrimination task without distractors

Similar to the perceptual task with distractors, a mixed ANOVA on *d′* prime was conducted on participants off their dopaminergic medication and healthy controls as between‐subject factors and foreperiod and validity as within‐subject factors. There were significant main effects of foreperiod and validity (Table S4), with no effect of group or an interaction between any of the factors (all *p*s > .3). Both groups had superior performance for targets appearing at the early interval, and both showed significant benefits of temporal orienting.

Moreover, there was no effect of dopaminergic medication on performance in this task. A repeated‐measures ANOVA on *d′*‐values for PD participants on and off medication revealed a significant main effect of foreperiod and validity (Table S7). There was no effect of medication or an interaction between any of the factors (all *ps* > .2). Moreover, there was a significant effect of validity and foreperiod in healthy control participants as well as PD participants on and off medication (Table S8).

Lastly, similar to perceptual discrimination task with distractors, we investigated the effect of group, medication and task conditions on response times. Tables S9 and S10 show that response times were similar for the PD and healthy‐control groups and that, overall, the PD group responded faster off medication compared to on medication regardless of task condition (see Tables S9 and S10).

In summary, similar to Experiment 1, while healthy control participants exhibited a perceptual advantage for validly cued information in the presence of distractors, this effect was abolished in PD participants off medication. This impairment was reversed in the same individuals when tested on their dopaminergic medication. Importantly, in the absence of distractors, PD participants on or off medication exhibited no impairment compared to healthy individuals.

#### 3.2.3 Perceptual discrimination task with and without distractors in patients on and off medication

To examine the effect of distractors on temporal cueing effects on PD participants on and off medication, we next performed a repeated‐measures ANOVA on *d*′ with foreperiod, validity, medication and task as within‐subject factors. There were significant main effects of foreperiod, validity and task (Table S11). Interestingly, the only significant interaction was observed between task and medication (*F*(1,13) = 4.89, *p* = .045, *η^2^* = .22); demonstrating an effect of medication only in the perceptual discrimination task with distractors (as reported above). For analysis of response times please see Table S12.

### 3.3 Discussion

In this study, we examined temporal orienting in individuals with PD. We revealed a striking dissociation in the effects of cued temporal orienting in tasks typically used to measure the benefits of temporally predictive cues to guide action versus perception. Whereas the benefits conferred by cued temporal orienting in a speeded‐response task were unaffected, its benefits for perceptual discrimination in a non‐speeded RSVP task were significantly compromised. At face value, the findings may have been suggestive of dissociable temporal orienting mechanisms for action versus perception. However, a further experiment showed that deficits in temporal orienting during perceptual discrimination were dependent on having to select a target stimulus from among temporally competing distractors. Overall the results indicate that cued temporal expectations are not critically dependent on the nigrostriatal system impaired in PD. However, specific task parameters may interact with the ability to utilize temporal expectations to guide adaptive behaviour. Our results also confirmed that healthy older adults can benefit from temporal cues to facilitate both motor responding and perceptual discrimination (Chauvin et al., 2016).

#### 3.3.1 Temporal orienting for motor responses

In line with previous neuropsychological studies in individuals with striatal lesions (Triviño et al., 2010) or with PD (Breska & Ivry, 2018; Mioni et al., 2018), we found that cued temporal orienting can be used to speed motor responses in individuals with PD. Our findings add to the evidence suggesting that the use of external timing signals to strengthen internalized expectations for motor responses does not critically depend on the basal ganglia. Relatedly, it has been shown that temporally predictable auditory signals can help improve walking and gait to become better synchronized (Bella et al., 2015; Benoit et al., 2014; Praamstra et al., 1998; te Woerd et al., 2014), remediate dysfunctional internal beat perception in music (Grahn & Brett, 2009), and improve speech perception (Kotz & Gunter, 2015; Kotz & Schmidt‐Kassow, 2015; Kotz et al., 2009). While the facilitation of motor responses by temporal orienting was unaffected in PD participants, they were still slower than control participants overall. This pattern of results is consistent with the proposal that the basal ganglia play an important role in coordinating motor actions (Braunlich & Seger, 2013). It is also consistent with the interpretation that basal‐ganglia deficits lead to a slowing of the internal clock (Ivry, 1996; Meck, 1998; Meck & Benson, 2002).

#### 3.3.2 Temporal orienting for perception

Compared with normal utilization of temporally predictive cues to guide speeded action, our results in Experiment 1 showed a surprising deficit in using these cues to guide perceptual discrimination. To emphasize perceptual discrimination, we used a non‐speeded version of an RSVP task, similar to what has been used before to emphasize perceptual, over motor, demands (Davranche et al., 2011). The results raised the possibility of dissociable systems for temporal orienting in the motor versus perceptual context, in which only the latter was critically dependent on the nigrostriatal system. An alternative explanation could be that the patterns of neural deficits in PD does not impair temporal orienting in general, but that the speeded‐response task was too simple and insensitive to pick up deficits in our cohort of PD participants. Still another possibility was that our non‐speeded RSVP task brought additional demands, other than perceptual load, that impeded the effective utilization of temporal orienting cues.

In addition to perceptual discrimination, the RSVP task requires inhibition of temporally distracting stimuli. The basal ganglia are considered important for regulating action through inhibitory mechanisms (Jenkinson & Brown, 2011), and their role in inhibition has also been proposed to extend to non‐motor functions (Fries, 2015; McNab & Klingberg, 2008; Womelsdorf & Fries, 2007). We considered, therefore, whether the basal ganglia could also contribute to inhibiting perceptual distractors within the context of an RSVP stream. The proposed role of the basal ganglia in timing together with a putative role in inhibiting perceptual competitors might therefore have combined to frustrate the ability to benefit from temporally predictive cues in our perceptual task.

Moreover, features of the perceptual‐discrimination task with distractors unrelated to temporal expectations may have prevented effects of temporal orienting to become manifest. The perceptual discrimination task with distractors may have required greater executive control in comparing incoming items to a working memory template to guide decision‐making. Both working‐memory and inhibitory functions have been associated with the basal ganglia and may have interacted to influence the effects observed in this task. As reviewed by McNab and Klingberg (2008), the basal ganglia may play an important role in allowing information to enter working memory, and previous research has highlighted the importance of the basal ganglia in sensory gating (Hazy et al., 2007; Schneider, 1984). It has further been proposed that top‐down control of attention can serve as a gatekeeper for working memory by biasing the encoding of information towards items that are most relevant (Chatham et al., 2014; McNab & Klingberg, 2008).

By stripping away the temporally competing stimuli from the non‐speeded task, while keeping the perceptual discrimination demands high, we obtained supportive evidence that the basal ganglia become critical when utilizing temporal cues to guide temporally precise selection of a target among proximal distractors. Whereas we replicated the deficits in utilizing temporally predictive cues in a non‐speeded perceptual discrimination task using RSVP in PD participants relative to well‐matched control participants, there was no difference between the participant groups for discriminating visual targets when no RSVP distractors were present (see also, Mioni et al., 2018).

We propose, therefore, that using temporal orienting for temporally gating perception and/or inhibiting distractors may be critically dependent on the nigrostriatal system or other areas compromised in PD. Since working memory capacity is predicted by prefrontal and basal ganglia activity (McNab & Klingberg, 2008), it is possible that PD‐related dysfunction of the basal ganglia is linked to the inability of our participants to inhibit distracting information effectively in the perceptual‐discrimination task to gate only the relevant stimuli for guiding subsequent performance.

One caveat to these interpretations, however, is that performance was lower overall in the task containing RSVP distractors, making it difficult to be certain that task difficulty did not contribute to the pattern of findings. In addition, it will be important to test the extent to which temporal orienting deficits in the context of temporal‐distractor inhibition may be associated to damage in other brain areas compromised in individuals with PD, such as frontal cortex. Studies combining behavioural testing with neural measures of structural or functional integrity will prove useful. Additionally, as demonstrated by the range in UPDRS scores, there is heterogeneity in our patient groups. Therefore, it is possible that performance in our tasks is differently influenced by different PD sub‐types, an open question that can be addressed in future studies using larger sample sizes. Such studies can also examine any possible changes in performance in temporal orienting of attention without any distractors as a result of dopaminergic medication.

The comparison of performance when PD participants were off versus on their medication showed that the ability to utilize temporally predictive cues when selecting targets among distractors was restored by medication. These findings suggest that the deficits off medication are not related to irreversible structural progressive deficits in PD, but rather were specific to the functioning of the nigrostriatal system whose activity can be at least partly reset through medication.

Overall, our results emphasize the importance of looking at interactions between temporal orienting and other task parameters. Temporal orienting does not occur in isolation; rather, it takes place within specific task demands and is frequently associated with other cognitive parameters such as those that involve working memory, other sources of selective attention (Rohenkohl et al., 2014), and decision‐making (Coull, 2014; Wiener et al., 2010). Future investigations should aim to disambiguate the conditions for temporal‐expectation impairments in neurological disorders, and determine the extent to which other factors, such as those related to task design, differences in cognitive abilities or patient heterogeneity, may interact with temporal orienting to explain differences in performance. Such considerations will be crucial in developing a thorough understanding of the neural systems and mechanisms necessary for utilizing temporal regularities to guide adaptive behaviour.

## CONFLICT OF INTEREST

We report no conflict of interest.

## AUTHOR CONTRIBUTIONS

Nahid Zokaei: conceptualization (supporting), data curation, software, investigation, formal analysis, visualization, writing – original draft (equal), writing – review & editing (lead). Celine R Gillebert: conceptualization (supporting), programming, investigation, formal analysis, visualization, writing – original draft (equal), writing – review & editing. Joshua J Chauvin: conceptualization (supporting), software, formal analysis, writing – review & editing. Daniela Gresch: investigation, writing – review & editing. Alexander G Board: investigation, writing – review & editing. Michal Rolinski: resources, writing – review & editing. Michele T Hu: resources, writing – review & editing. Anna C Nobre: conceptualization (lead), funding acquisition, supervision, writing – original draft preparation (equal), writing – review & editing.

### PEER REVIEW

The peer review history for this article is available at https://publons.com/publon/10.1111/ejn.15114.

## Supporting information

Table S1‐S12

## Data Availability

The conditions of our ethics approval do not permit public archiving of the data supporting this study, and sharing of data requires a formal data‐sharing agreement in accordance with ethical procedures governing the re‐use of sensitive data. Readers seeking access to the data should contact the first author.
