# Supplementary material for: Temporal orienting in Parkinson’s disease
Source: Eur J Neurosci. 2021 Feb 2;53(8):2713–25. doi: 10.1111/ejn.15114 (PMC8290223; doi:10.1111/ejn.15114)
Supplement: Supplementary file 1 — Table S1‐S12 [file EJN-53-2713-s001.docx]

**Supplementary Material**

**Table 1**. Experiment 1. Analyses of variance (ANOVAs) on reaction time (ms) values from the speeded-response task.

| **Effect** | **df1** | **df2** | ***F*** | ***p*** | ***η^2^*** |
| --- | --- | --- | --- | --- | --- |
| **Mixed ANOVA with participant group as between-subjects factor** | | | | | |
| Group* | 1 | 33 | 6.28 | <.05 | .16 |
| Foreperiod* | 1 | 33 | 50.51 | <.001 | .61 |
| Foreperiod x Group | 1 | 33 | .19 | .67 |  |
| Validity* | 1 | 33 | 48.16 | <.001 | .59 |
| Validity x Group | 1 | 33 | .42 | .55 |  |
| Foreperiod x Validity* | 1 | 33 | 17.19 | <.001 | .34 |
| Foreperiod x Validity x Group | 1 | 33 | 1.70 | .20 |  |
| **Repeated measures ANOVA in control participants** | | | | | |
| Foreperiod* | 1 | 16 | 46.47 | <.001 | .74 |
| Validity* | 1 | 16 | 54.41 | <.001 | .77 |
| Foreperiod x Validity* | 1 | 16 | 17.48 | <.001 | .52 |
| **Repeated measures ANOVA in participants with Parkinson’s disease** | | | | | |
| Foreperiod* | 1 | 17 | 19.48 | <.001 | .53 |
| Validity* | 1 | 17 | 18.42 | <.001 | .52 |
| Foreperiod x Validity* | 1 | 17 | 8.84 | <.01 | .34 |
|  |  |  |  |  |  |

*Note.* * = significant effects

**Table 2.** Experiment 1. Analyses of variance (ANOVAs) on *d’*-values from the perceptual-discrimination task.

| Effect | df1 | df2 | *F* | *p* | *η^2^* |
| --- | --- | --- | --- | --- | --- |
| **Mixed ANOVA with participant group as between-subjects factor** | | | | | |
| Group | 1 | 34 | 3.13 | .09 |  |
| Foreperiod* | 1 | 34 | 13.20 | <.001 | .28 |
| Foreperiod x Group | 1 | 34 | .15 | .70 |  |
| Validity* | 1 | 34 | 19.16 | <.001 | .36 |
| Validity x Group* | 1 | 34 | 4.65 | <.05 | .12 |
| Foreperiod x Validity | 1 | 34 | 1.01 | .32 |  |
| Foreperiod x Validity x Group | 1 | 34 | 2.08 | .16 |  |
| **Repeated measures ANOVA in control participants** | | | | | |
| Foreperiod* | 1 | 17 | 4.48 | <.05 | .21 |
| Validity* | 1 | 17 | 21.21 | <.001 | .56 |
| Foreperiod x Validity | 1 | 17 | 12.53 | .13 |  |
| **Repeated measures ANOVA in participants with Parkinson’s disease** | | | | | |
| Foreperiod* | 1 | 17 | 9.83 | <.01 | .37 |
| Validity | 1 | 17 | 2.48 | .13 |  |
| Foreperiod x Validity | 1 | 17 | .18 | .74 |  |

*Note.* * = significant effect

**Table 3.** Experiment 2. Analyses of variance (ANOVAs) on *d’*-values from the perceptual-discrimination task with distractors.

| Effect | df1 | df2 | F | P | *η^2^* |
| --- | --- | --- | --- | --- | --- |

**Mixed ANOVA with participant group as between-subject factor**

**PD participants off medication vs. Healthy controls**

| Group | 1 | 30 | .6 | .5 |  |
| --- | --- | --- | --- | --- | --- |
| Foreperiod* | 1 | 30 | 6.19 | .019 | .17 |
| Foreperiod x Group | 1 | 30 | .86 | .3 |  |
| Validity* | 1 | 30 | 5.86 | .022 | .16 |
| Validity x Group* | 1 | 30 | 4.54 | .041 | .13 |
| Foreperiod x Validity | 1 | 30 | .13 | .7 |  |
| Foreperiod x Validity x Group | 1 | 30 | .6 | .5 |  |

**Mixed ANOVA with participant group as between-subject factor**

**PD participants on medication vs. Healthy controls**

| Group | 1 | 30 | .04 | .84 |  |
| --- | --- | --- | --- | --- | --- |
| Foreperiod* | 1 | 30 | 10.42 | .003 | .26 |
| Foreperiod x Group | 1 | 30 | .63 | .43 |  |
| Validity* | 1 | 30 | 13.7 | .001 | .31 |
| Validity x Group | 1 | 30 | .06 | .8 |  |
| Foreperiod x Validity | 1 | 30 | .41 | .53 |  |
| Foreperiod x Validity x Group | 1 | 30 | .52 | .5 |  |

**Repeated measures ANOVA in PD participants on and off medication**

| Medication | 1 | 13 | 1.04 | .3 |  |
| --- | --- | --- | --- | --- | --- |
| Foreperiod | 1 | 13 | 2.67 | .13 |  |
| Foreperiod x Medication | 1 | 13 | .003 | .96 |  |
| Validity* | 1 | 13 | 6.63 | .023 | .34 |
| Validity x Medication* | 1 | 13 | 12.2 | 007 | .44 |
| Foreperiod x Validity | 1 | 13 | 0 | .99 |  |
| Foreperiod x Validity x Medication | 1 | 13 | .16 | .7 |  |

*Note.* * = significant effects

**Table 4.** Experiment 2. Analyses of variance (ANOVAs) on *d’*-values from the perceptual-discrimination task with distractors per each participant group.

| Effect | df1 | df2 | F | P | *η^2^* |
| --- | --- | --- | --- | --- | --- |

**Repeated measures ANOVA in control participants**

| Foreperiod* | 1 | 17 | 7.21 | .016 | .29 |
| --- | --- | --- | --- | --- | --- |
| Validity* | 1 | 17 | 8.08 | .011 | .32 |
| Foreperiod x Validity | 1 | 17 | .6 | .4 |  |

**Repeated measures ANOVA in PD participants off medication**

| Foreperiod | 1 | 13 | .98 | .3 |  |
| --- | --- | --- | --- | --- | --- |
| Validity | 1 | 13 | .1 | .7 |  |
| Foreperiod x Validity | 1 | 13 | .11 | .7 |  |

**Repeated measures ANOVA in PD participants on medication**

| Foreperiod | 1 | 13 | 2.15 | .16 |  |
| --- | --- | --- | --- | --- | --- |
| Validity* | 1 | 13 | 10.5 | .006 | .45 |
| Foreperiod x Validity | 1 | 13 | .066 | .8 |  |

*Note.* * = significant effects

**Table 5.** Experiment 2. Analyses of variance (ANOVAs) on response times from the perceptual-discrimination task with distractors.

| Effect | df1 | df2 | F | P | *η^2^* |
| --- | --- | --- | --- | --- | --- |

**Mixed ANOVA with participant group as between-subject factor**

**PD participants off medication vs. Healthy controls**

| Group | 1 | 30 | .4 | .5 |  |
| --- | --- | --- | --- | --- | --- |
| Foreperiod | 1 | 30 | 3.99 | .055 |  |
| Foreperiod x Group | 1 | 30 | .53 | .47 |  |
| Validity* | 1 | 30 | 20.4 | <.001 | .4 |
| Validity x Group | 1 | 30 | 4.54 | .041 |  |
| Foreperiod x Validity | 1 | 30 | .26 | .62 |  |
| Foreperiod x Validity x Group | 1 | 30 | .8 | .4 |  |

**Mixed ANOVA with participant group as between-subject factor**

**PD participants on medication vs. Healthy controls**

| Group | 1 | 30 | .1.3 | .27 |  |
| --- | --- | --- | --- | --- | --- |
| Foreperiod | 1 | 30 | 2.8 | .1 |  |
| Foreperiod x Group | 1 | 30 | 1.44 | .24 |  |
| Validity* | 1 | 30 | 20.9 | <.001 | .41 |
| Validity x Group | 1 | 30 | .5 | .5 |  |
| Foreperiod x Validity | 1 | 30 | 1.41 | .25 |  |
| Foreperiod x Validity x Group | 1 | 30 | .005 | .9 |  |

**Repeated-measures ANOVA in PD participants on and off medication**

| Medication | 1 | 13 | 5.6 | .035 | 0.03 |
| --- | --- | --- | --- | --- | --- |
| Foreperiod | 1 | 13 | .96 | .34 |  |
| Foreperiod x Medication | 1 | 13 | .022 | .65 |  |
| Validity* | 1 | 13 | 10.26 | .007 | .44 |
| Validity x Medication* | 1 | 13 | 0 | .99 |  |
| Foreperiod x Validity | 1 | 13 | .35 | .56 |  |
| Foreperiod x Validity x Medication | 1 | 13 | .54 | .5 |  |

*Note.* * = significant effects

**Table 6.** Experiment 2. Analyses of variance (ANOVAs) on response times from the perceptual-discrimination task with distractors per each participant group.

| Effect | df1 | df2 | F | P | *η^2^* |
| --- | --- | --- | --- | --- | --- |

**Repeated measures ANOVA in control participants**

| Foreperiod | 1 | 17 | 3.68 | .07 |  |
| --- | --- | --- | --- | --- | --- |
| Validity* | 1 | 17 | 12.5 | .003 | .42 |
| Foreperiod x Validity | 1 | 17 | .8 | .4 |  |

**Repeated measures ANOVA in PD participants off medication**

| Foreperiod | 1 | 13 | .98 | .3 |  |
| --- | --- | --- | --- | --- | --- |
| Validity* | 1 | 13 | 8.4 | .013 | .4 |
| Foreperiod x Validity | 1 | 13 | .13 | .7 |  |

**Repeated measures ANOVA in PD participants on medication**

| Foreperiod | 1 | 13 | .16 | .7 |  |
| --- | --- | --- | --- | --- | --- |
| Validity* | 1 | 13 | 8.75 | .011 | .4 |
| Foreperiod x Validity | 1 | 13 | .64 | .4 |  |

*Note.* * = significant effects

**Table 8.** Experiment 2. Analyses of variance (ANOVAs) on *d’*-values from the perceptual-discrimination task without distractors per each participant group.

| Effect | df1 | df2 | F | P | *η^2^* |
| --- | --- | --- | --- | --- | --- |

**Repeated measures ANOVA in control participants**

| Foreperiod* | 1 | 17 | 25.2 | <.001 | .59 |
| --- | --- | --- | --- | --- | --- |
| Validity* | 1 | 17 | 19.9 | <.001 | .54 |
| Foreperiod x Validity | 1 | 17 | .35 | .56 |  |

**Repeated measures ANOVA in PD participants off medication**

| Foreperiod* | 1 | 13 | 9.4 | .009 | .42 |
| --- | --- | --- | --- | --- | --- |
| Validity* | 1 | 13 | 23.4 | <.001 | .64 |
| Foreperiod x Validity | 1 | 13 | .76 | .4 |  |

**Repeated measures ANOVA in PD participants on medication**

| Foreperiod* | 1 | 13 | 4.86 | .046 | .27 |
| --- | --- | --- | --- | --- | --- |
| Validity* | 1 | 13 | 20.9 | .001 | .62 |
| Foreperiod x Validity | 1 | 13 | .14 | .7 |  |

*Note.* * = significant effects

**Table 9.** Experiment 2. Analyses of variance (ANOVAs) on response times from the perceptual-discrimination task without distractors.

| Effect | df1 | df2 | F | P | *η^2^* |
| --- | --- | --- | --- | --- | --- |

**Mixed ANOVA with participant group as between-subject factor**

**PD participants off medication vs. Healthy controls**

| Group | 1 | 30 | .17 | .7 |  |
| --- | --- | --- | --- | --- | --- |
| Foreperiod | 1 | 30 | .19 | .67 |  |
| Foreperiod x Group | 1 | 30 | 1.07 | .3 |  |
| Validity* | 1 | 30 | 86 | <.001 | .74 |
| Validity x Group | 1 | 30 | 0 | .99 |  |
| Foreperiod x Validity | 1 | 30 | 1.87 | .18 |  |
| Foreperiod x Validity x Group | 1 | 30 | 2.7 | .11 |  |

**Mixed ANOVA with participant group as between-subject factor**

**PD participants on medication vs. Healthy controls**

| Group | 1 | 30 | 1.7 | .2 |  |
| --- | --- | --- | --- | --- | --- |
| Foreperiod* | 1 | 30 | 7.68 | .009 | .2 |
| Foreperiod x Group | 1 | 30 | .97 | .33 |  |
| Validity* | 1 | 30 | 81.9 | <.001 | .73 |
| Validity x Group | 1 | 30 | . 5 | .5 |  |
| Foreperiod x Validity* | 1 | 30 | 8.44 | .007 | .22 |
| Foreperiod x Validity x Group | 1 | 30 | .8 | .38 |  |

**Repeated measures ANOVA in PD participants on and off medication**

| Medication* | 1 | 13 | 6.34 | .026 | .33 |
| --- | --- | --- | --- | --- | --- |
| Foreperiod | 1 | 13 | 1.2 | .29 |  |
| Foreperiod x Medication | 1 | 13 | 1.97 | .18 |  |
| Validity* | 1 | 13 | 57 | <.001 | .82 |
| Validity x Medication | 1 | 13 | 1.27 | .28 |  |
| Foreperiod x Validity | 1 | 13 | .3 | .59 |  |
| Foreperiod x Validity x Medication | 1 | 13 | 1.48 | .25 |  |

*Note.* * = significant effects

**Table 10.** Experiment 2. Analyses of variance (ANOVAs) on response times from the perceptual-discrimination task without distractors per each participant group.

| Effect | df1 | df2 | F | P | *η^2^* |
| --- | --- | --- | --- | --- | --- |

**Repeated measures ANOVA in control participants**

| Foreperiod | 1 | 17 | 2.67 | .12 |  |
| --- | --- | --- | --- | --- | --- |
| Validity* | 1 | 17 | 41 | <.001 | .71 |
| Foreperiod x Validity | 1 | 17 | 6.9 | .018 | .29 |

**Repeated measures ANOVA in PD participants off medication**

| Foreperiod | 1 | 13 | .09 | .76 |  |
| --- | --- | --- | --- | --- | --- |
| Validity* | 1 | 13 | 51.9 | <.001 | .8 |
| Foreperiod x Validity | 1 | 13 | .03 | .87 |  |

**Repeated measures ANOVA in PD participants on medication**

| Foreperiod | 1 | 13 | 4.4 | .055 |  |
| --- | --- | --- | --- | --- | --- |
| Validity* | 1 | 13 | 40.78 | <.001 | .76 |
| Foreperiod x Validity | 1 | 13 | 2.4 | .14 |  |

*Note.* * = significant effects

**Table 11.** Experiment 2. Analyses of variance (ANOVAs) on *d’*-values from the perceptual-discrimination task with and without distractors in patients with PD on and off medication

| Effect | df1 | df2 | F | P | *η^2^* |
| --- | --- | --- | --- | --- | --- |

**Repeated measures ANOVA with medication status as between-subject factor**

**PD participants off and on medication**

| Medication | 1 | 13 | 3.65 | .078 |  |
| --- | --- | --- | --- | --- | --- |
| Foreperiod* | 1 | 13 | 7.34 | .018 | .36 |
| Validity* | 1 | 13 | 27.5 | <.001 | .68 |
| Task* | 1 | 13 | 37.5 | <.001 | .74 |
| Foreperiod x Validity | 1 | 13 | .86 | .37 |  |
| Foreperiod x Task | 1 | 13 | 3.12 | .1 |  |
| Validity x Task | 1 | 13 | 3.91 | .07 |  |
| Foreperiod x Validity x Task | 1 | 13 | .51 | .49 |  |
| Foreperiod x Medication | 1 | 13 | 1.38 | .26 |  |
| Medication x Validity | 1 | 13 | 1.98 | .18 |  |
| Foreperiod x Validity x Medication | 1 | 13 | .051 | .83 |  |
| Task x Medication* | 1 | 13 | 4.89 | .045 | .27 |
| Foreperiod x Task x Medication | 1 | 13 | 2.21 | .161 |  |
| Task x Validity x Medication | 1 | 13 | 3.41 | .09 |  |
| Foreperiod x Validity x Task x Medication | 1 | 13 | .45 | .84 |  |

*Note.* * = significant effects

**Table 12.** Experiment 2. Analyses of variance (ANOVAs) on response times from the perceptual-discrimination task with and without distractors in patients with PD on and off medication

| Effect | df1 | df2 | F | P | *η^2^* |
| --- | --- | --- | --- | --- | --- |

**Repeated measures ANOVA with medication status as between-subject factor**

**PD patients off and on medication**

| Medication* | 1 | 13 | 33 | <.001 | .72 |
| --- | --- | --- | --- | --- | --- |
| Foreperiod | 1 | 13 | 1.73 | .21 |  |
| Validity* | 1 | 13 | 136 | <.001 | .91 |
| Task* | 1 | 13 | 9.15 | .01 | .41 |
| Foreperiod x Validity* | 1 | 13 | 4.95 | .044 | .27 |
| Foreperiod x Task | 1 | 13 | .001 | .97 |  |
| Validity x Task | 1 | 13 | 8.3 | .013 | .39 |
| Foreperiod x Validity x Task | 1 | 13 | 2.77 | .12 |  |
| Foreperiod x Medication* | 1 | 13 | 9.5 | .009 | .42 |
| Medication x Validity | 1 | 13 | .93 | .35 |  |
| Foreperiod x Validity x Medication | 1 | 13 | 1.59 | .23 |  |
| Task x Medication | 1 | 13 | 2.5 | .14 |  |
| Foreperiod x Task x Medication* | 1 | 13 | 6.12 | .028 | .32 |
| Task x Validity x Medication | 1 | 13 | .94 | .35 |  |
| Foreperiod x Validity x Task x Medication | 1 | 13 | .13 | .73 |  |

*Note.* * = significant effects
